# Hypoxic preconditioned MSCs-derived small extracellular vesicles for photoreceptor protection in retinal degeneration

**DOI:** 10.1186/s12951-023-02225-2

**Published:** 2023-11-25

**Authors:** Yuntong Sun, Yuntao Sun, Shenyuan Chen, Yifan Yu, Yongjun Ma, Fengtian Sun

**Affiliations:** 1 Department of Clinical Laboratory, Affiliated Jinhua Hospital, Zhejiang University School of Medicine, Jinhua, 321000 Zhejiang China; 2 Jiangsu Province Key Laboratory of Medical Science and Laboratory Medicine, Department of Laboratory Medicine, School of Medicine, Jiangsu University, Zhenjiang, 212013 Jiangsu China

**Keywords:** Photoreceptor apoptosis, Retinal degeneration, Small extracellular vesicles, Mesenchymal stem/stromal cells, Hypoxia, GAP43

## Abstract

Photoreceptor apoptosis is an important pathogenesis of retinal degeneration and a primary cause of vision loss with limited treatment methods. Mesenchymal stem/stromal cells-derived small extracellular vesicles (MSC-sEVs) have shown therapeutic value in various ocular disorders. Recent studies have revealed that hypoxic preconditioning can improve the effectiveness of MSC-sEVs in tissue regeneration. However, whether hypoxic preconditioned MSC-sEVs (Hyp-sEVs) exert superior effects on photoreceptor protection relative to normoxic conditioned MSC-sEVs (Nor-sEVs) remains unclear. Here, we reported that Hyp-sEVs further improved retinal structure, recovered retinal function, and suppressed photoreceptor apoptosis in N-methyl-N-nitrosourea (MNU)-induced mouse model compared with Nor-sEVs. Hyp-sEVs also exhibited enhanced anti-apoptotic roles in MNU-provoked 661 W cell injury in vitro. We then analyzed the protein profiles of Nor-sEVs and Hyp-sEVs by LC-MS/MS and found that growth-associated protein 43 (GAP43) was enriched in Hyp-sEVs. The knockdown of GAP43 abolished the retinal therapeutic effects of Hyp-sEVs. Mechanistically, hypoxic stimulation-induced hypoxia-inducible factor-1α (HIF-1α) activation was responsible for preventing tripartite motif-containing protein 25 (TRIM25)-mediated GAP43 ubiquitination and degradation, leading to the upregulation of GAP43 in Hyp-sEVs. Together, our findings uncover the efficacy and mechanism of Hyp-sEVs-based photoreceptor protection and highlight the potential of Hyp-sEVs as optimized therapeutics for retinal degeneration.

**Graphical abstract:**

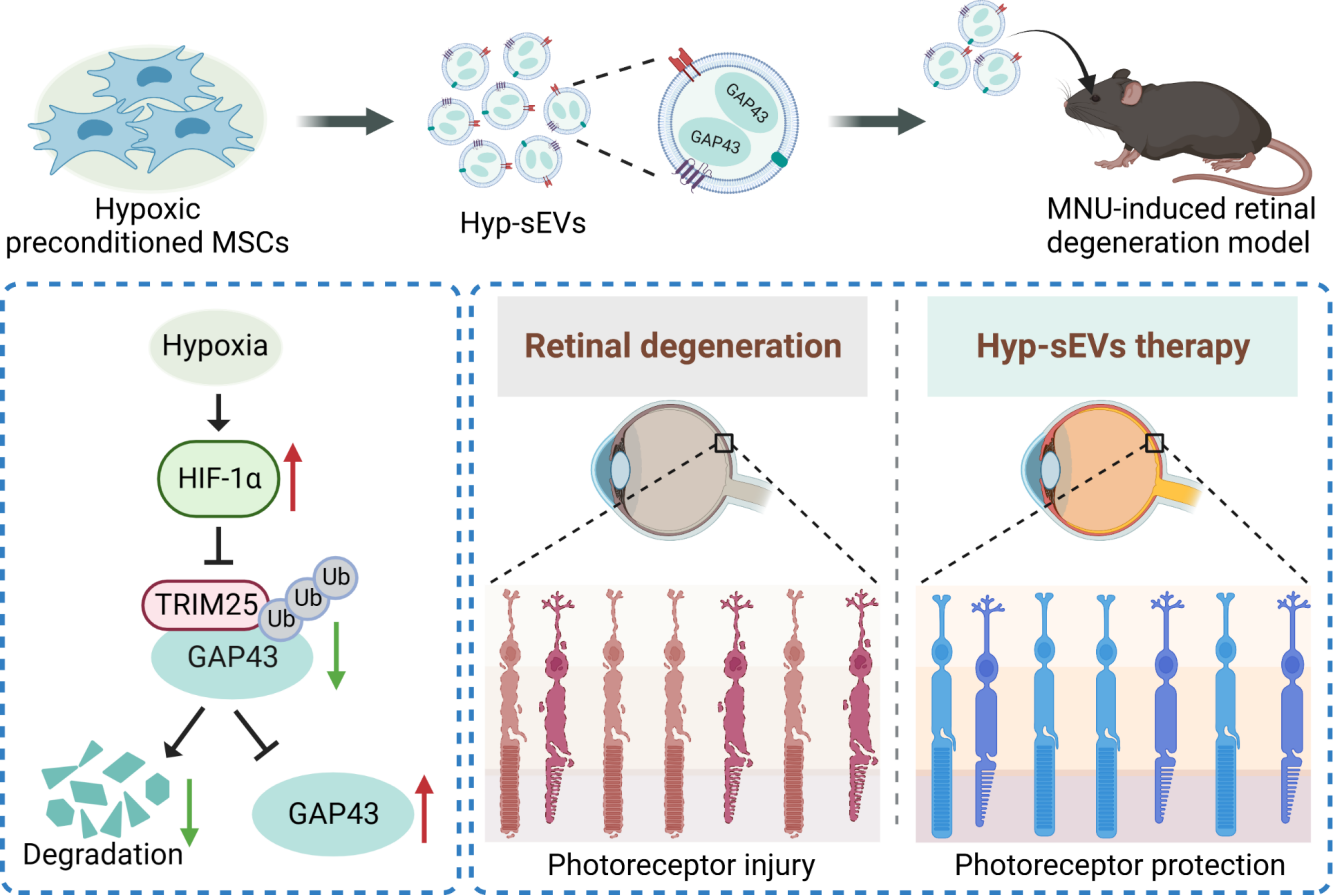

**Supplementary Information:**

The online version contains supplementary material available at 10.1186/s12951-023-02225-2.

## Background

The progressive photoreceptor loss is a major cause of irreversible visual decline and blindness in retinal degenerative disorders such as retinitis pigmentosa [1, 2]. Multiple factors-induced photoreceptor apoptosis has been recognized as the crucial contributor to the pathogenesis of these diseases [3]. Current clinical and experimental strategies mainly include gene therapy, anti-apoptotic agents, retinal transplantation, and stem cell therapy [4, 5]. However, the treatment efficacy and overall prognosis remain unsatisfactory. The establishment of novel therapeutics to rescue photoreceptor apoptosis in retinal degeneration is urgently required.

As a type of nano-sized membranous vesicles, small extracellular vesicles (sEVs) mediate the intercellular communication and participate in the regulation of various physiological and pathological processes by transporting proteins, nucleic acids, and lipids [6, 7]. Emerging evidence has revealed that the release of sEVs represents the main therapeutic mechanism underlying mesenchymal stem/stromal cell (MSC) transplantation [8]. The intravitreal injection of MSC-derived sEVs (MSC-sEVs) is considered as a promising cell-free approach to treat ocular diseases. MSC-sEVs can reduce the incidence of dry age-related macular degeneration by regulating Nrf2/Keap1 signaling pathway [9]. Notably, treatment with MSC-sEVs also inhibits photoreceptor loss and recovers retinal function in degenerative retinopathies [10]. However, there are still many inherent limitations including the replicative senescence of MSC-induced decreased therapeutic potential and low yield that restrict the clinical application of MSC-sEVs in tissue regeneration [11]. Growing studies have suggested that oxygen tension serves as an important factor that modulates the biological function of MSCs [12]. The increased beneficial effects of hypoxic preconditioned MSC-sEVs (Hyp-sEVs) for various diseases such as myocardial infarction, diabetic ulcer and spinal cord injury have attracted widespread attention [13–15]. Nevertheless, whether Hyp-sEVs exhibit enhanced therapeutic roles to ameliorate photoreceptor apoptosis and improve retinal degeneration has not yet been evaluated.

In this study, the alkylating agent N-methyl-N-nitrosourea (MNU) which can specifically cause photoreceptor cell loss was used to establish the mouse model of retinal degeneration and induce 661 W photoreceptor cell injury in vitro. We observed that Hyp-sEVs showed elevated therapeutic efficiency to protect photoreceptors against apoptosis in counteracting retinal degeneration compared with normoxic conditioned MSC-sEVs (Nor-sEVs). Mass spectrometry and gene knockdown analysis showed that growth-associated protein 43 (GAP43) was upregulated in Hyp-sEVs and mediated the retinal therapeutic effects of Hyp-sEVs. Furthermore, we also revealed that hypoxic preconditioning-induced hypoxia-inducible factor-1α (HIF-1α) activation prevented tripartite motif-containing protein 25 (TRIM25)-mediated GAP43 ubiquitination and degradation in MSCs, leading to the enrichment of GAP43 in Hyp-sEVs. These findings suggest that Hyp-sEVs administration serves as a novel strategy for retinal degeneration therapy.

## Materials and methods

### Cell culture, identification, and treatment

MSCs were isolated from the fresh human umbilical cords as described previously [16]. After the permission of mothers, human umbilical cords were placed in phosphate buffer solution (PBS) containing penicillin-streptomycin for 30 min, followed by the removal of arteries and veins. Subsequently, the umbilical cords were cut into 2-cm pieces, pasted on the bottom of culture dishes, placed upside-down in the culture incubator for 60 min, and maintained in serum-free DMEM (Life Technologies, USA) at 37 °C with 5% CO_2_. The culture medium was changed every 3 days. Primary cells were trypsinized and passaged for further expansion. MSCs at passage 3 were cultured in normoxic (21% O_2_) or hypoxic (1% O_2_) conditions at 37 °C with 5% CO_2_ respectively. Normoxic conditioned MSCs (Nor-MSCs) and hypoxic preconditioned MSCs (Hyp-MSCs) were then seeded in osteogenic medium (Cyagen Biosciences, USA) and adipogenic medium (Cyagen Biosciences, USA) for 2 weeks, and stained with Alizarin Red S and Oil Red O to detect their differentiation potential. Flow cytometry assay was used to detect the markers of Nor-MSCs and Hyp-MSCs including CD29, CD44, CD73, CD11b, CD34, and CD45.

The 661 W cells were purchased from the Chinese Academy of Sciences (Shanghai, China) and have been characterized previously to be of cone photoreceptor cell lineage [17]. The 661 W cells were maintained in high-glucose DMEM (Bioind, Israel) with 10% fetal bovine serum (FBS), 100 U/ml penicillin, and 100 g/ml streptomycin at 37 °C with 5% CO_2_. In vitro treatment of 661 W cells with MNU (Sigma-Aldrich, USA) was conducted at the concentration of 300 µg/ml for 6 h, followed by the administration of sEVs (20 µg/mL) for 24 h.

### Isolation and identification of sEVs

sEVs were isolated from the cell supernatant of MSCs cultured with serum-free medium by ultracentrifugation. Briefly, the conditioned medium was centrifuged at 300 × g for 15 min, 2,000 × g for 20 min and 10,000 × g for 30 min to remove cells and cell debris. The clarified supernatant was then ultracentrifuged at 100,000 × g for 90 min to obtain sEVs precipitation. After washing with PBS, the sEVs were ultracentrifuged at 100,000 × g for 90 min, dissolved with PBS, passed through a 0.22-mm filter, and stored at -80 °C. The morphology of sEVs was observed by the transmission electron microscopy (TEM). The size distribution and particle number of sEVs were analyzed by NanoSight tracking analysis (NTA). The expressions of protein markers including CD9, CD63, Alix, tumor susceptibility gene 101 (TSG101), and endoplasmic reticulum protein calnexin were detected by Western blot.

### Animal model and treatment

All animal experiments were approved by the Medical Ethics Committee and Ethics Committee for Experimental Animals of Jiangsu University (2,020,161). The 12-week-old C57BL/6 male mice were purchased from the Animal Center of Jiangsu University, and housed in a pathogen-free environment with a 12 h light/dark cycle at the temperature of 25 °C with free access to food and water. For the establishment of MNU-induced retinal degeneration model, mice were intraperitoneally injected with MNU (50 mg/kg in sterile saline). The collected sEVs were adjusted to the protein concentration of 1 µg/µl and injected at 1 µl into each vitreous chamber at 6 h after MNU treatment with a 33-G Hamilton syringe. All mice at 7 d after MNU treatment were sacrificed with 4% isoflurane and the eyeballs were collected for further analysis. The number of mice used in each experiment is indicated in the figure legend.

### Tracing of sEVs

The membrane fluorescent dye PKH26 (5 µM) was incubated with Nor-sEVs and Hyp-sEVs for 30 min at 37 °C. Subsequently, the stained Nor-sEVs and Hyp-sEVs were washed with PBS and centrifuged at 100,000 g for 90 min. For the retinal tracing of sEVs, the retinal tissues were isolated at 24 h after the intravitreal injection of PKH26-labeled Nor-sEVs and Hyp-sEVs, fixed in 4% paraformaldehyde, cryoprotected with 30% sucrose, embedded in the OCT compound, and cut into 15-µm retinal sections. After the counterstaining with Hoechst 33,342 (Sigma-Aldrich, USA), retinal sections were observed under a confocal microscope (DeltaVision Elite, GE, USA). For in vitro tracing, 661 W cells were treated with PKH26-labeled Nor-sEVs and Hyp-sEVs for 24 h, fixed in 4% paraformaldehyde, counterstained with Hoechst 33,342 (Sigma-Aldrich, USA), and observed under a confocal microscope (DeltaVision Elite, GE, USA).

### Electroretinogram (ERG) analysis

After overnight dark adaptation, mice were anesthetized by the intraperitoneal injection with ketamine (87.5 mg/kg) and xylazine (12.5 mg/kg), and the cornea was anesthetized with 0.5% proxymetacaine. The ground electrodes, reference electrodes and record electrodes were then placed on the tail, mouth, and cornea respectively. The flash intensity was 3.0 cd.s/m^2^. The UTAS Visual Diagnostic System (LKC Technologies, USA) was used to record the scotopic and photopic responses for analyzing the a-wave and b-wave.

### Hematoxylin and eosin (H&E) staining

After enucleation, retinal tissues were rapidly isolated, fixed in 4% paraformaldehyde overnight, embedded in paraffin, and cut into 4-µm sections. Retinal sections were stained with H&E for the observation of retinal morphology according to the standard protocols.

### Immunofluorescence staining

After deparaffinization, retinal sections were treated with boiled citrate buffer (pH 6.0, 10 mM) to repair antigens, blocked with 5% bovine serum albumin (BSA) for 1 h and incubated with the primary antibody at 4 °C overnight. The 661 W cells were fixed with 4% paraformaldehyde on the slide for 30 min, permeabilized with 0.1% Triton X-100 for 15 min, blocked with 5% BSA for 1 h and incubated with the primary antibody at 4 °C overnight. Subsequently, the sections were stained with fluorescent-conjugated secondary antibody for 1 h at 37 °C, counterstained with Hoechst 33,342 (Sigma-Aldrich, USA), and observed under a confocal microscope (DeltaVision Elite, GE, USA). The primary antibodies included S-opsin (1:100, ABN1660, Millipore, USA), Ki-67 (1:100, 34,330, CST, USA), Cleaved Caspase-3 (1:100, 9661, CST, USA), and GAP43 (1:100, 16971-1-AP, Proteintech, China).

### Terminal deoxynucleotidyl transferase dUTP nick end labelling (TUNEL) staining

TUNEL staining was performed according to the manufacturer’s instructions of TUNEL BrightRed Apoptosis Detection Kit (Vazyme, China). Briefly, each section was incubated with 100 µL of proteinase K solution for 20 min, balanced in 100 µL of 1 × equilibration buffer for 30 min, treated with 100 µL of TDT buffer for 1 h at 37 °C to label apoptotic cells, and counterstained with Hoechst 33,342 (Sigma-Aldrich, USA). After washing with PBS, the sections were photographed using a confocal microscope (DeltaVision Elite, GE, USA).

### Liquid chromatography-tandem mass spectrometry (LC-MS/MS)

50 µg of each protein sample was subjected to digestion with the sequencing-grade trypsin, followed by the peptide labeling. The peptides were analyzed by LC-MS/MS. Multiple databases were used for the functional annotation analysis of identified proteins. Correlation analysis on differentially expressed proteins was also performed.

### Transfection of siRNA and plasmid

Specific targeting siRNA and overexpressing plasmid were designed and synthesized by Genepharma (Suzhou, China). The full-length cDNA of the human HIF-1α gene was inserted into the pCDH-CMV-MCS-GFP + Puro plasmid vector to construct the overexpression plasmid. Cells were resuspended and seeded in 6-well plates (2 × 10^5^ cells/well). When the cell density reached 50–70%, cells were transfected with siRNA and plasmid using Lipofectamine 2000 (Life Technologies, USA) in serum-free medium according to the manufacturer’s instructions. At 6 h after transfection, cells were exposed to the fresh complete medium and cultured for another 24 h. The sequences of siRNA were as follows:

siRNA NC: sense: UUCUCCGAACGUGUCACGUTT.

siRNA NC: antisense: ACGUGACACGUUCGGAGAATT.

si-HIF-1α: sense: CUCCCUAUAUCCCAAUGGATT.

si-HIF-1α: antisense: UCCAUUGGGAUAUAGGGAGTT.

### Lentiviral knockdown of GAP43 in Hyp-MSCs

The lentiviral vectors containing GAP43 short hairpin RNA (shRNA) sequence and negative control vector were designed and synthesized by Genepharma (Suzhou, China). Recombinant lentiviruses were produced by the transfection in HEK293T cells using Lipofectamine 2000 (Life Technologies, USA). Hyp-MSCs were then transfected with recombinant lentivirus. The stable cell lines were cultured in serum-free medium for 48 h to obtain supernatants, followed by the isolation of sEVs by ultracentrifugation. Western blot was used to evaluate the efficiency of GAP43 knockdown in Hyp-MSCs and Hyp-sEVs. The sequences of shRNA were as follows:

Control shRNA: CCTAAGGTTAAGTCGCCCTCG.

GAP43 shRNA: GCTCATAAGGCCGCAACCAAA.

### Western blot

The total protein of retinas, cells and sEVs was isolated using radio-immunoprecipitation assay lysis buffer. The protein concentration was determined using the BCA assay kit. Equal amounts of protein samples were separated through 12% SDS-PAGE gel and transferred onto PVDF membranes (Millipore, USA). After blocking in 5% skim milk for 1 h, the membranes were incubated with primary antibodies at 4 °C overnight, treated with HRP-conjugated secondary antibodies at 37 °C for 1 h, and detected by enhanced chemiluminescence. The primary antibodies included Oct4 (1:1000, 2750, CST, USA), Sall4 (1:1000, 8459, CST, USA), Lin28a (1:1000, 11724-1-AP, Proteintech, China), Sox2 (1:1000, 3579, CST, USA), TSG101 (1:1000, 72,312, CST, USA), Alix (1:1000, 92,880, CST, USA), CD9 (1:1000, 98,327, CST, USA), CD63 (1:2000, 52,090, CST, USA), calnexin (1:1000, 2433, CST, USA), proliferating cell nuclear antigen (PCNA; 1:1000, 10205-2-AP, Proteintech, China), Bcl-2 (1:500, 12789-1-AP, Proteintech, China), Bax (1:500, 50599-2-Ig, Proteintech, China), GAP43 (1:1000, 16971-1-AP, Proteintech, China), HIF-1α (1:1000, 36,169, CST, USA), Ubiquitin (1:2000, 58,395, CST, USA), TRIM25 (1:1000, 13,773, CST, USA), and β-actin (1:5000, 4970, CST, USA).

### Co-immunoprecipitation (Co-IP) assay

Cells were lysed in Co-IP buffer and incubated with the GAP43 antibody (1:100, 16971-1-AP, Proteintech, China) at 4 °C overnight, followed by the treatment with magnetic beads for 4 h. After washing with Co-IP buffer, protein complexes were detected by western blot.

### qRT-PCR

The total RNA of cells was isolated using Trizol reagent (Gibco, USA) and reverse-transcribed to cDNA according to the manufacturer’s protocol of HiScript II 1st Strand cDNA Synthesis Kit (Vazyme, China). Based on the ABI Prism 2720 PCR system, qRT-PCR was performed with SYBR Green PCR kit (CWBIO, China). The relative mRNA expression was evaluated using the 2^−ΔΔCt^ method. The sequences of primers were as follows:

β-actin: forward: GACCTGTACGCCAACACAGT.

β-actin: reverse: CTCAGGAGGAGCAATGATCT.

GAP43: forward: GGCCGCAACCAAAATTCAGG.

GAP43: reverse: CGGCAGTAGTGGTGCCTTC.

### CCK8 assay

After treatment, cells were resuspended and seeded in 96-well plates (3,000 cells/well). After the incubation for 24, 48, 72 and 96 h, cells were treated with 100 µL of fresh medium containing 10 µL of CCK8 reagent (Vazyme, China) for 3 h. The absorbance of each well was determined at 450 nm using the spectrophotometer (FLX800, BioTek, USA).

### Cell apoptosis assay

Annexin V–FITC/PI Apoptosis Detection Kit (Vazyme, China) was used to assess the effect of sEVs on cell apoptosis. After the treatment with MNU and sEVs, 661 W cells were washed with PBS, centrifuged at 800 rpm for 5 min, resuspended in 1× binding buffer, and stained with 5 µl of Annexin V-FITC and 5 µl of PI at room temperature. The stained cells were then detected by flow cytometry (FACSCalibur, BD, USA). The percentages of early apoptotic (Annexin V^+^PI^−^) plus late apoptotic (Annexin V^+^PI^+^) cells were considered as apoptotic percentages.

### Statistical analysis

All statistical analysis was performed using GraphPad Prism software (GraphPad, San Diego, USA). All data were presented as the means ± SEM. Unpaired Student’s t-test was used for comparison between two groups. The significant differences among multiple groups were assessed by analysis of variance with Newman-Keuls test. *P* value < 0.05 was considered statistically significant.

## Results

### Hypoxic preconditioning enhanced the cellular viability and paracrine effect of MSCs

**Fig. 1:**
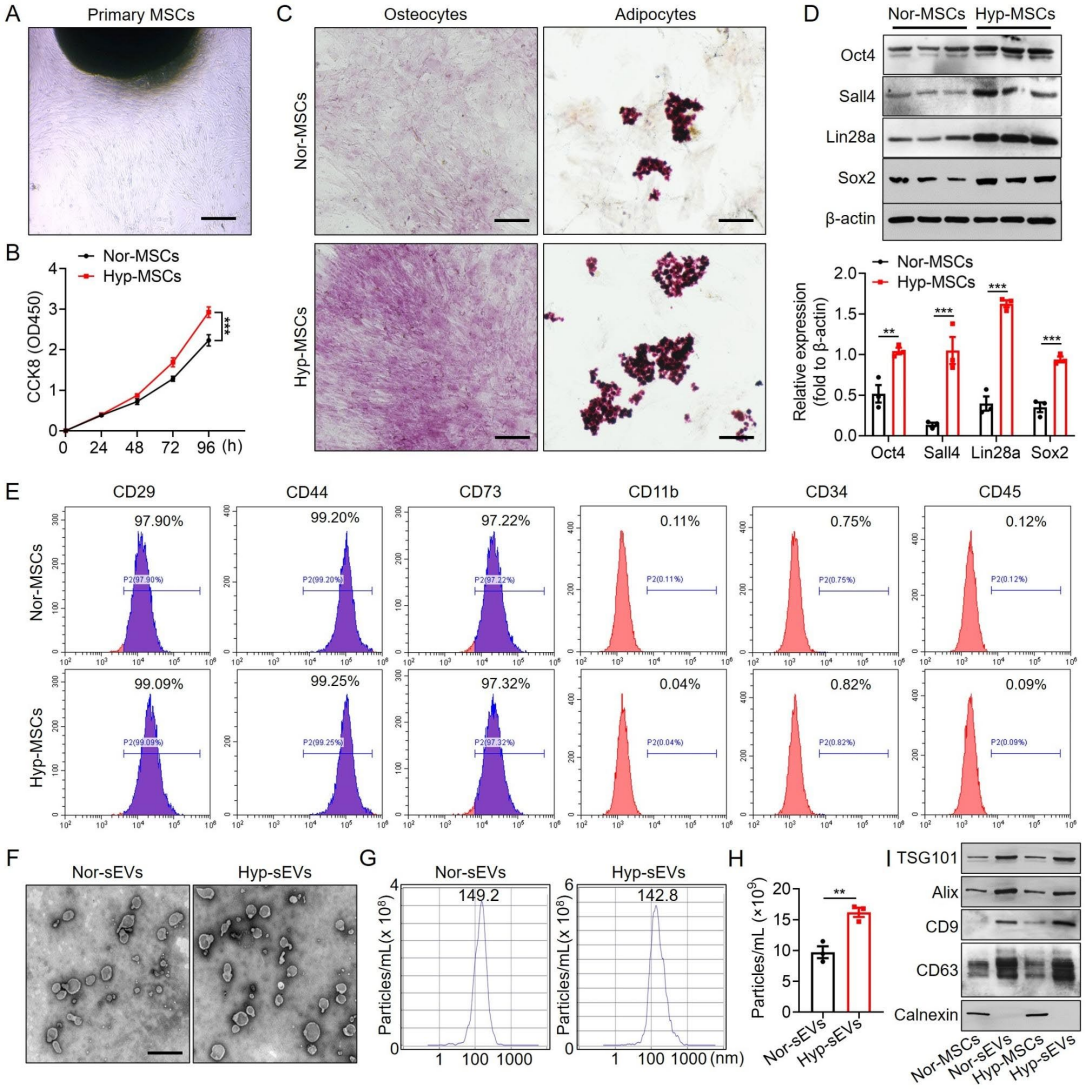
Characterization of MSCs and sEVs. (**A**) The morphological observation of primary MSCs. Scale bars, 100 μm. (**B**) CCK8 assay for the proliferation of Nor-MSCs and Hyp-MSCs (n = 5). (**C**) Alizarin Red S staining and Oil Red O staining for detecting the osteogenic and adipogenic differentiation of Nor-MSCs and Hyp-MSCs. Scale bars, 100 μm. (**D**) Western blot for the stemness-related indicators of Nor-MSCs and Hyp-MSCs. (**E**) Flow cytometry for the phenotypic markers of Nor-MSCs and Hyp-MSCs. (**F**) TEM images of Nor-sEVs and Hyp-sEVs. Scale bars, 100 nm. (**G**) NTA for the size distribution and particle number of Nor-sEVs and Hyp-sEVs. (**H**) The particle number comparation of Nor-sEVs and Hyp-sEVs (n = 3). (**I**) Western blot for the protein markers of Nor-sEVs and Hyp-sEVs. All data are presented as means ± SEM. **P < 0.01 and ***P < 0.001

Based on the previously established separation method, MSCs were isolated from human umbilical cords (Fig. 1A). To investigate the effect of hypoxic preconditioning on cellular function, MSCs at passage 3 were cultured in normoxic or hypoxic conditions respectively. CCK8 assay showed that hypoxic stimulation promoted the proliferation of MSCs (Fig. 1B). After osteogenic and adipogenic medium inductions, the typical calcium nodule structure and red lipid droplets were observed in MSCs (Fig. 1C). Compared with Nor-MSCs group, Hyp-MSCs exhibited enhanced multidirectional differentiation potential. Western blot result demonstrated that hypoxic preconditioning upregulated the expressions of stemness-related indicators in MSCs including Oct4, Sall4, Lin28a, and Sox2 (Fig. 1D). Moreover, flow cytometry assay revealed that Nor-MSCs and Hyp-MSCs positively expressed CD29, CD44 and CD73, and negatively expressed CD11b, CD34 and CD45 (Fig. 1E). Subsequently, we purified sEVs from the conditioned medium of Nor-MSCs and Hyp-MSCs. The results of TEM and NTA showed that Nor-sEVs and Hyp-sEVs exhibited the cup-shaped morphology with an average diameter of less than 150 nm, while the particle number of Hyp-sEVs was markedly higher than Nor-sEVs (Fig. 1F-H). Western blot analysis demonstrated that Nor-sEVs and Hyp-sEVs both expressed sEVs-associated proteins including TSG101, Alix, CD9, and CD63, whereas the endoplasmic reticulum protein calnexin was not detected (Fig. 1I). These findings suggested that hypoxic preconditioning could enhance the cellular activity of MSCs and promote the secretion of sEVs.

### Enhanced therapeutic effects of Hyp-sEVs on MNU-induced retinal degeneration and photoreceptor injury

**Fig. 2:**
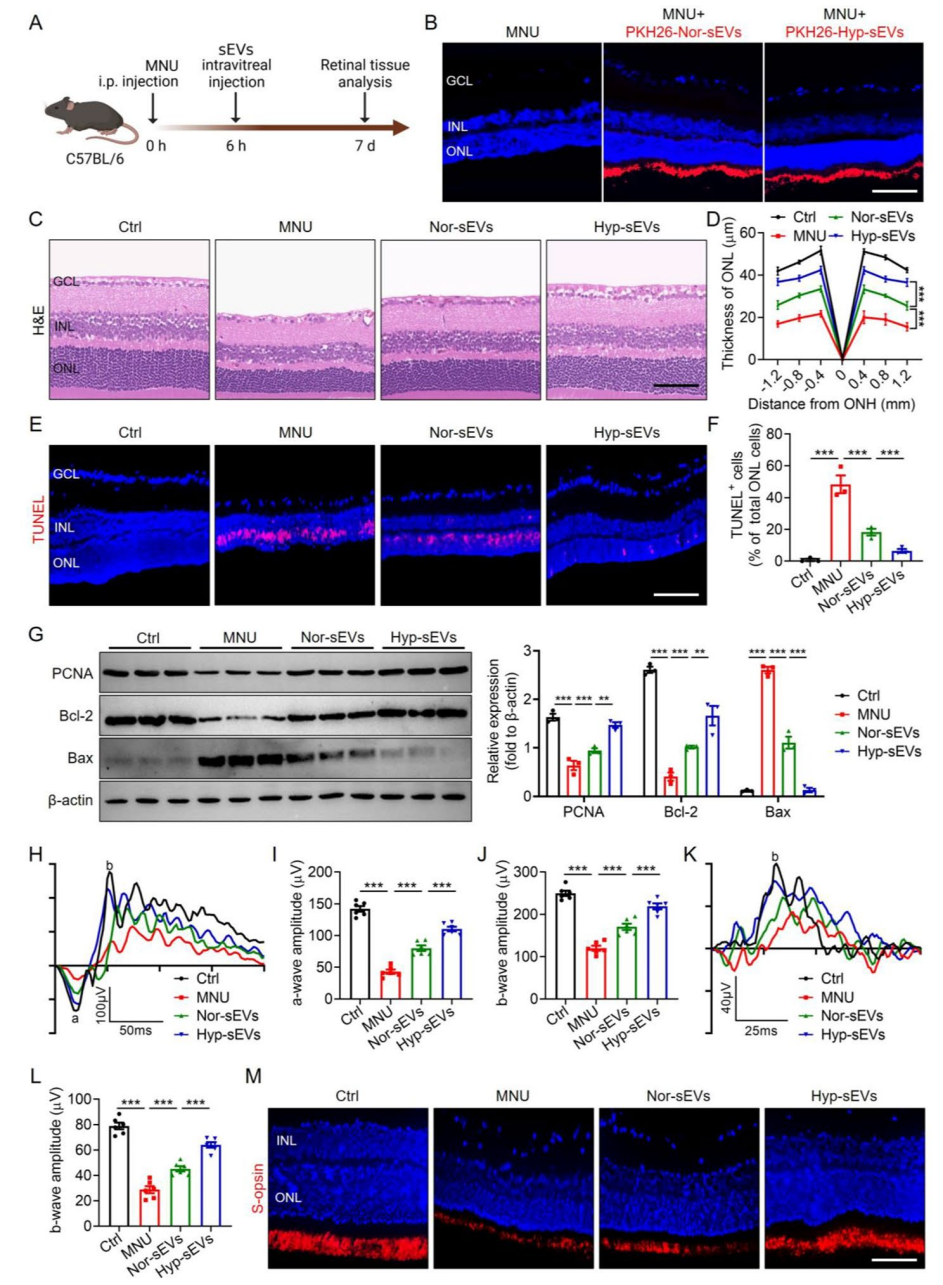
Enhanced therapeutic effects of Hyp-sEVs on MNU-induced photoreceptor injury. (**A**) Schematic diagram demonstrating the study design of in vivo experiments. (**B**) Tracing of PKH26-labeled sEVs in retina tissues of MNU mice after intravitreal injection for 24 h. Scale bars, 50 μm. (**C**, **D**) H&E staining of retinal tissues and the corresponding quantitative analysis of ONL thickness (n = 3). Scale bars, 50 μm. (**E**, **F**) TUNEL staining of retinal tissues and the corresponding quantitative analysis of TUNEL^+^ cell percentage of total ONL cells (n = 3). Scale bars, 50 μm. (**G**) Western blot for the retinal expressions of PCNA, Bcl-2 and Bax (n = 3). (**H**-**J**) Representative scotopic ERG waveforms and the corresponding quantitative analysis of scotopic a-wave and b-wave amplitude changes (n = 6). (**K**, **L**) Representative photopic ERG waveforms and the corresponding quantitative analysis of photopic b-wave amplitude change (n = 6). (**M**) Retinal immunofluorescent staining of S-opsin. Scale bars, 25 μm. All data are presented as means ± SEM. **P < 0.01 and ***P < 0.001. GCL, ganglion cell layer; INL, inner nuclear layer; ONL, outer nuclear layer

To investigate whether Hyp-sEVs treatment serves as a more effective strategy for photoreceptor protection in retinal degeneration, the phototoxin MNU was used to establish the photoreceptor-specific injury model in mice, followed by the intravitreal injection of Nor-sEVs and Hyp-sEVs (Fig. 2A). Biodistribution analysis revealed that Nor-sEVs and Hyp-sEVs were distributed in various retinal layers of normal mice after injection (Fig. S1), whereas Nor-sEVs and Hyp-sEVs were specifically located in the photoreceptor-nuclei-residing outer nuclear layer (ONL) of MNU-treated mice (Fig. 2B). H&E staining of retinal tissues showed that the intraperitoneal injection of MNU resulted in the retinal degeneration and ONL loss, whereas Nor-sEVs treatment alleviated MNU-induced retinal structural damage, and Hyp-sEVs could further maintain retinal integrity and improve ONL thickness (Fig. 2C, D). Retinal TUNEL staining also revealed the Hyp-sEVs-mediated enhanced inhibition of photoreceptor apoptosis in the ONL compared with Nor-sEVs group (Fig. 2E, F). The results of Western blot presented that Hyp-sEVs remarkably upregulated the expressions of PCNA and Bcl-2 and downregulated the expression of Bax in retinal tissues of MNU-induced mouse model (Fig. 2G). Subsequently, we tested the photoreceptor function by ERG analysis including scotopic and photopic responses, which reflect the hyperpolarization of photoreceptors and depolarization of bipolar cells postsynaptic to photoreceptors. Compared with Nor-sEVs group, Hyp-sEVs exerted more effective roles to protect retinal function, quantified by the increased amplitudes of dark-adapted scotopic a- and b-waves as well as the light-adapted photopic b-wave (Fig. 2H-L). S-opsin staining also showed that Hyp-sEVs further alleviated MNU-induced loss of cone photoreceptors (Fig. 2M). These findings indicated that Hyp-sEVs exhibited elevated therapeutic effects on MNU-trigged retinal photoreceptor injury relative to Nor-sEVs.

### Hyp-sEVs inhibited MNU-induced apoptosis of 661 w cells

**Fig. 3:**
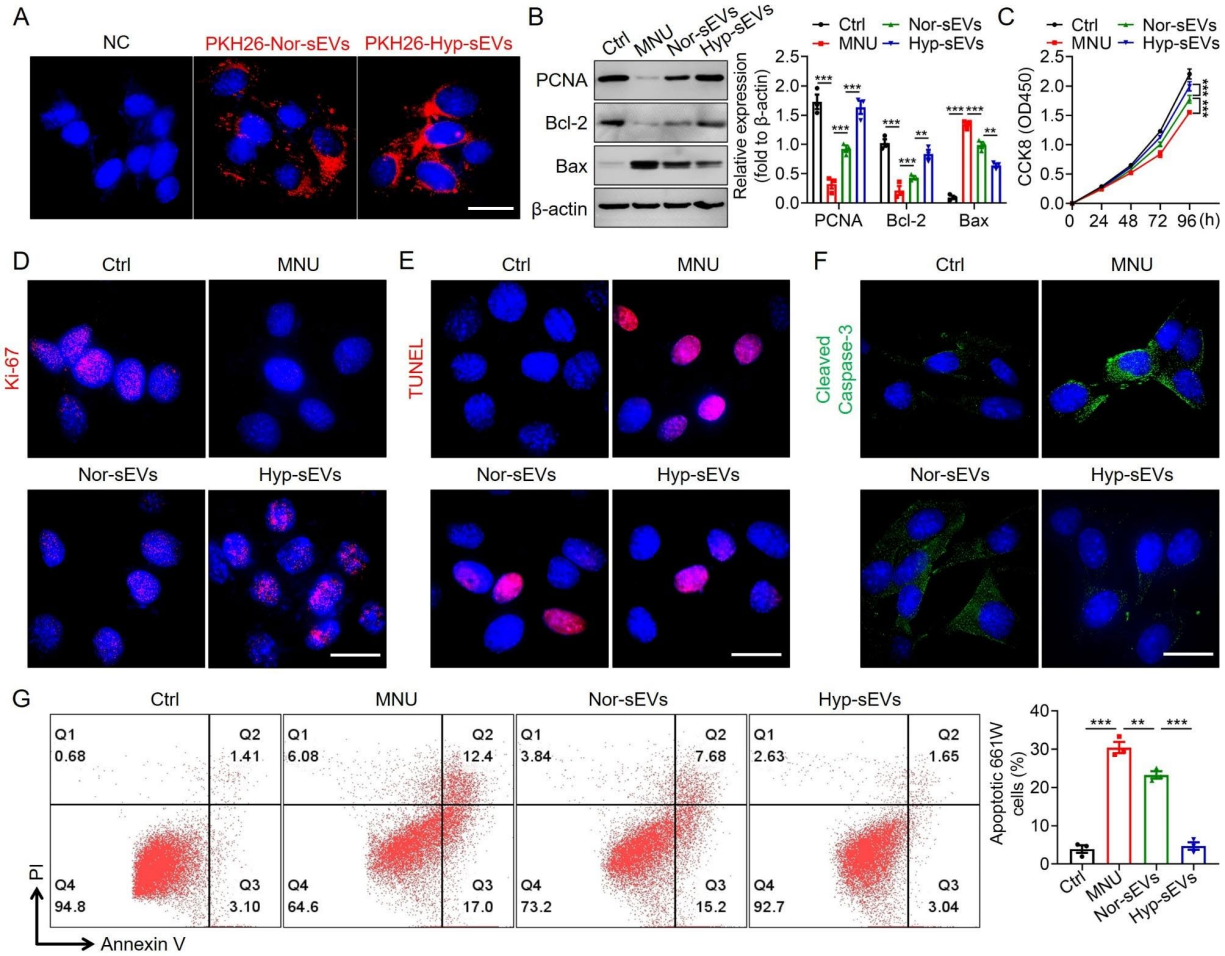
The enhanced anti-apoptotic role of Hyp-sEVs in MNU-treated 661 W cells. (**A**) The uptake of PKH26-labeled sEVs by 661 W cells after co-incubation for 24 h. Scale bars, 25 μm. (**B**) Western blot for the expressions of PCNA, Bcl-2 and Bax in 661 W cells. (**C**) CCK8 assay for the proliferation of 661 W cells (n = 5). (**D**) Immunofluorescent staining images of 661 W cells showing Ki-67 expression. Scale bars, 25 μm. (**E**) TUNEL staining of 661 W cells. Scale bars, 25 μm. (**F**) Immunofluorescent staining images of 661 W cells showing Cleaved Caspase-3 expression. Scale bars, 25 μm. (**G**) Cell apoptosis assay of 661 W cells (n = 3). All data are presented as means ± SEM. **P < 0.01 and ***P < 0.001

In vitro, 661 W cells treated with MNU were used as the cell model, and then administrated with Nor-sEVs and Hyp-sEVs respectively. The tracing experiment confirmed the internalization of Nor-sEVs and Hyp-sEVs by 661 W cells after co-incubation (Fig. 3A). The results of Western blot showed that Hyp-sEVs could further reversed the MNU-induced downregulation of PCNA and Bcl-2 and upregulation of Bax compared with Nor-sEVs (Fig. 3B). CCK8 assay and Ki-67 staining demonstrated that Hyp-sEVs efficiently promoted the proliferation of 661 W cells treated with MNU (Fig. 3C, D). Moreover, the results of TUNEL staining, Cleaved caspase-3 staining, and cell apoptosis assay also presented that Hyp-sEVs treatment exhibited an enhanced inhibition on MNU-induced apoptosis of 661 W cells relative to Nor-sEVs group (Fig. 3E-G). These data suggested that Hyp-sEVs exerted more effective anti-apoptotic roles than Nor-sEVs in vitro.

### The enrichment of GAP43 in Hyp-sEVs

**Fig. 4:**
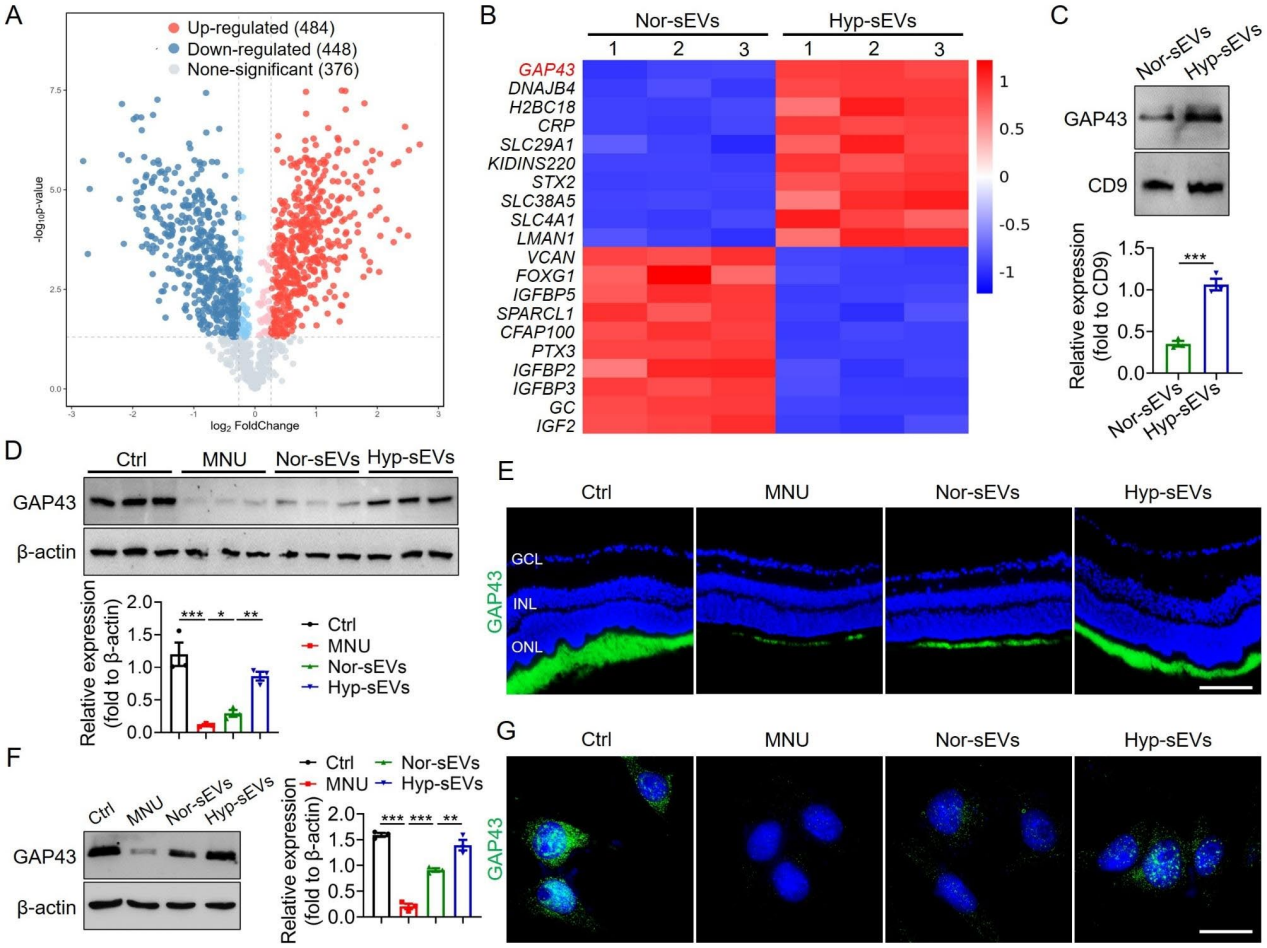
Hyp-sEVs-mediated delivery of GAP43. (**A**, **B**) LC-MS/MS analysis for the differentially expressed proteins in Nor-sEVs and Hyp-sEVs. (**C**) Western blot for the expression of GAP43 in Nor-sEVs and Hyp-sEVs. (**D**) Western blot for the expression of GAP43 in retinal tissues (n = 3). (**E**) Immunofluorescent staining images of retinal tissues showing GAP43 expression. Scale bars, 50 μm. (**F**) Western blot for the expression of GAP43 in 661 W cells. (**G**) Immunofluorescent staining images of 661 W cells showing GAP43 expression. Scale bars, 25 μm. All data are presented as means ± SEM. *P < 0.05, **P < 0.01, and ***P < 0.001

To explore the major components that mediate the retinal therapeutic effect of Hyp-sEVs, we analyzed the protein profile of Hyp-sEVs by LC-MS/MS. Compared with Nor-sEVs, approximately 484 proteins were enriched in Hyp-sEVs (Fig. 4A). Among them, the upregulation of GAP43 was the most significant (Fig. 4B). Thus, we hypothesized whether Hyp-sEVs alleviated MNU-induced retinal photoreceptor damage by delivering GAP43. We verified that the expression of GAP43 in Hyp-sEVs was higher than that in Nor-sEVs (Fig. 4C). Moreover, the results of Western blot and immunofluorescence staining showed that Hyp-sEVs administration remarkably promoted GAP43 accumulation in retinal tissues of MNU-treated mice (Fig. 4D, E). Consistently, the measurement of GAP43 in 661 W cells demonstrated that Hyp-sEVs further restored the MNU-induced downregulation of GAP43 compared with Nor-sEVs group (Fig. 4F, G).

### GAP43 knockdown attenuated the anti-apoptotic effects of Hyp-sEVs in vitro

**Fig. 5:**
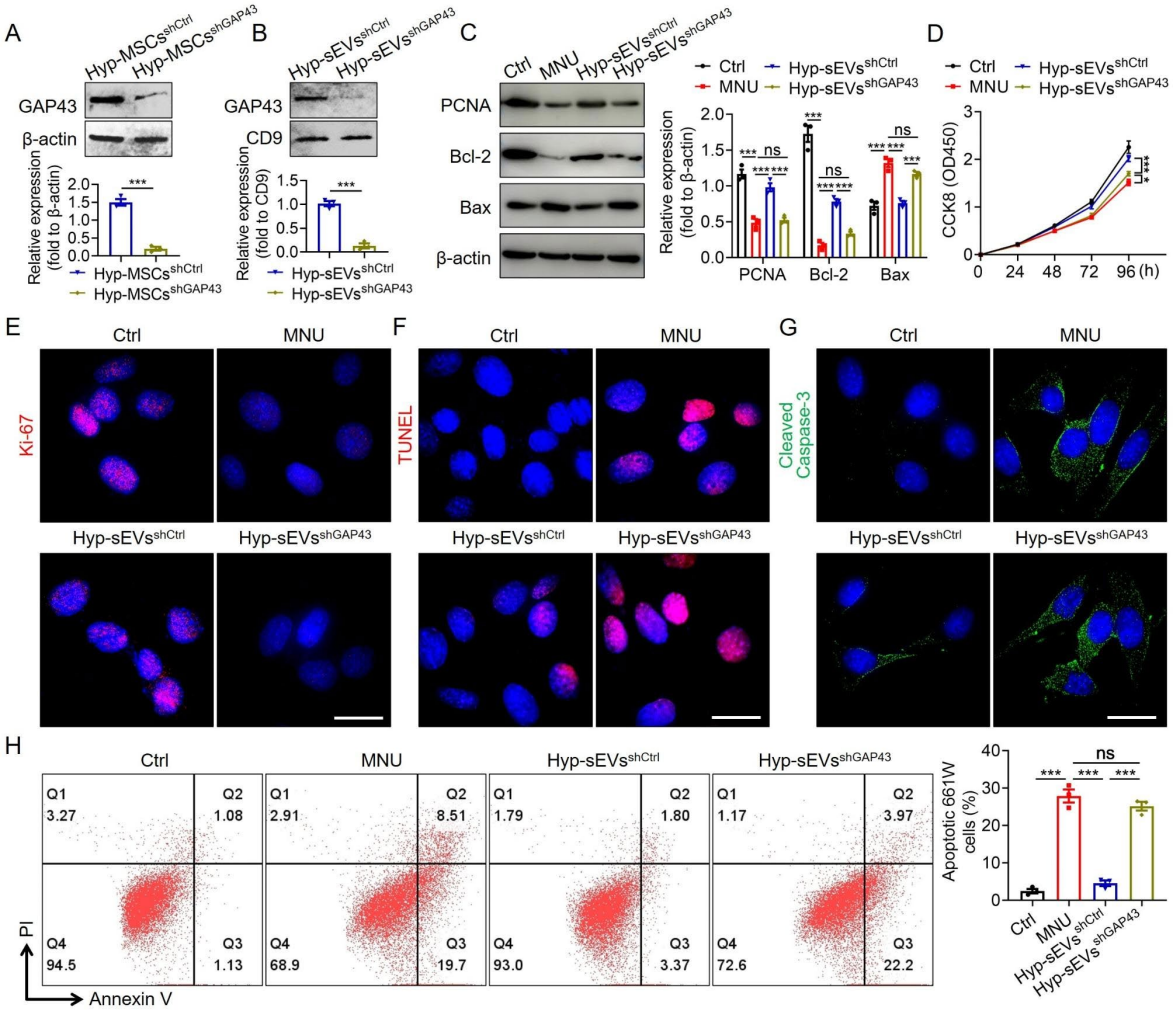
GAP43 mediated the protection of Hyp-sEVs on MNU-induced apoptosis of 661 W cells. (**A**, **B**) Western blot for the knockdown efficiency of GAP43 in Hyp-MSCs and Hyp-sEVs after transfection. (**C**) Western blot for the expressions of PCNA, Bcl-2 and Bax in 661 W cells. (**D**) CCK8 assay for the proliferation of 661 W cells (n = 5). (**E**) Immunofluorescent staining images of 661 W cells showing Ki-67 expression. Scale bars, 25 μm. (**F**) TUNEL staining of 661 W cells. Scale bars, 25 μm. (**G**) Immunofluorescent staining images of 661 W cells showing Cleaved Caspase-3 expression. Scale bars, 25 μm. (**H**) Cell apoptosis assay of 661 W cells (n = 3). All data are presented as means ± SEM. ns, not significant, *P < 0.05, and ***P < 0.001

To determine the role of GAP43 in Hyp-sEVs-mediated retinal therapy, we knocked down GAP43 expression in Hyp-MSCs with lentivirus carrying specific shRNA. Western blot analysis confirmed the effective knockdown of GAP43 in GAP43 shRNA-transfected Hyp-MSCs (Hyp-MSCs^shGAP43^) (Fig. 5A). Subsequently, we isolated Hyp-MSCs^shGAP43^-derived sEVs (Hyp-sEVs^shGAP43^) from the conditioned medium of Hyp-MSCs^shGAP43^ and found that the expression of GAP43 was markedly reduced in Hyp-sEVs^shGAP43^ relative to control group (Fig. 5B). Hyp-sEVs-mediated the upregulation of PCNA and Bcl-2 and downregulation of Bax in MNU-treated 661 W cells were reversed by GAP43 knockdown (Fig. 5C). The results of CCK8 assay and Ki-67 staining also showed that Hyp-sEVs^shGAP43^ exerted little effects to promote the proliferation of 661 W cells (Fig. 5D, E). Moreover, GAP43 knockdown resulted in Hyp-sEVs treatment failing to reduce TUNEL-positive cells, decrease Cleaved Caspase-3 expression, and inhibit the apoptosis of 661 W cells (Fig. 5F-H). These data revealed that GAP43 was an important factor for Hyp-sEVs-mediated anti-apoptotic activity in vitro.

### Hyp-sEVs-delivered GAP43 prevented MNU-induced retinal degeneration and photoreceptor injury in vivo

**Fig. 6:**
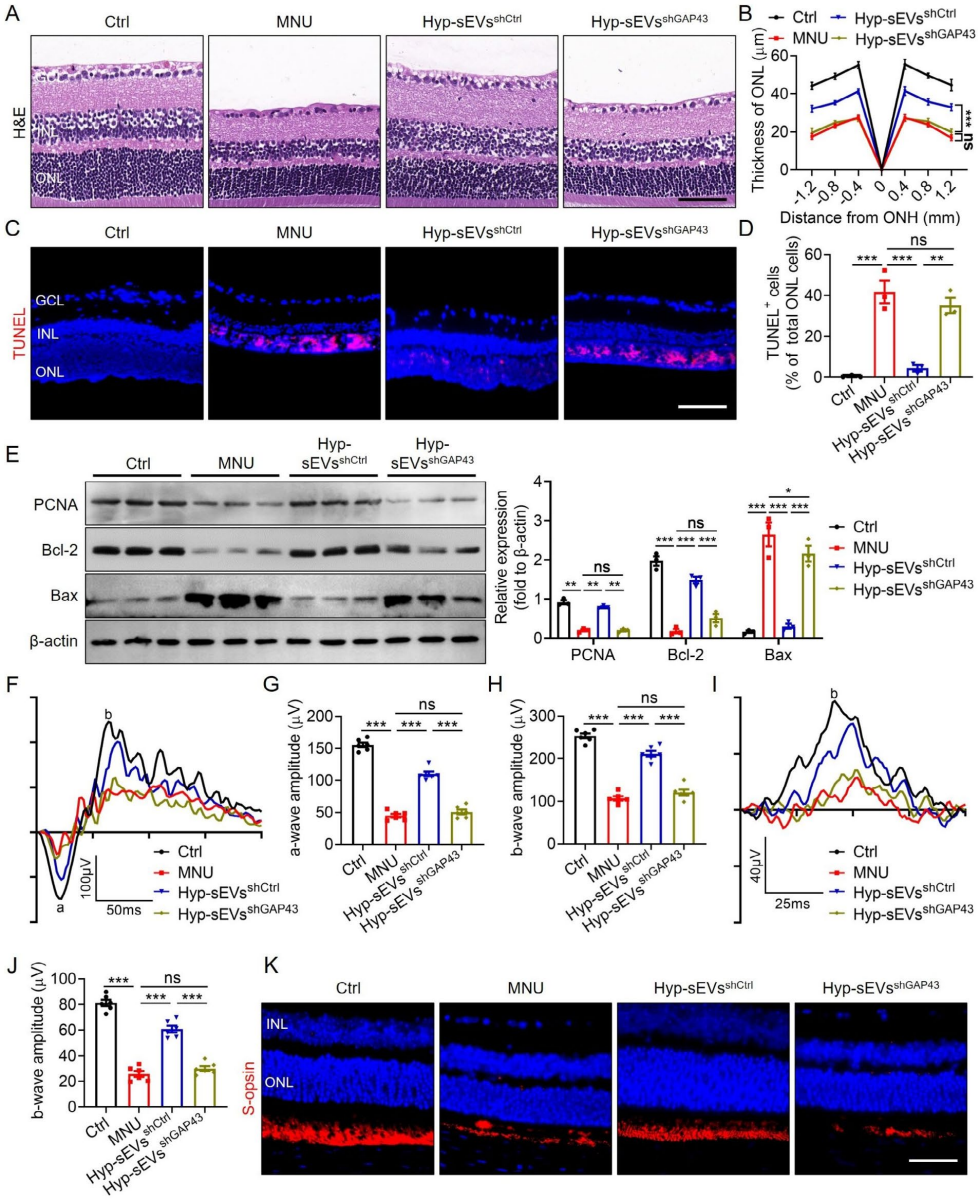
Hyp-sEVs alleviated MNU-induced retinal photoreceptor injury by delivering GAP43. (**A**, **B**) H&E staining of retinal tissues and the corresponding quantitative analysis of ONL thickness (n = 3). Scale bars, 50 μm. (**C**, **D**) TUNEL staining of retinal tissues and the corresponding quantitative analysis of TUNEL^+^ cell percentage of total ONL cells (n = 3). Scale bars, 50 μm. (**E**) Western blot for the retinal expressions of PCNA, Bcl-2 and Bax (n = 3). (**F**-**H**) Representative scotopic ERG waveforms and the corresponding quantitative analysis of scotopic a-wave and b-wave amplitude changes (n = 6). (**I**, **J**) Representative photopic ERG waveforms and the corresponding quantitative analysis of photopic b-wave amplitude change (n = 6). (**K**) Retina immunofluorescent staining of S-opsin. Scale bars, 25 μm. All data are presented as means ± SEM. ns, not significant, *P < 0.05, **P < 0.01 and ***P < 0.001. GCL, ganglion cell layer; INL, inner nuclear layer; ONL, outer nuclear layer

Next, we evaluated the essential role of GAP43 in Hyp-sEVs-mediated retinal photoreceptor protection in vivo. H&E staining of retinal sections showed that GAP43 knockdown abolished the Hyp-sEVs-induced preservation of retinal structure and improvement of ONL thickness (Fig. 6A, B). The results of TUNEL staining and Western blot also showed that Hyp-sEVs^shctrl^, but not Hyp-sEVs^shGAP43^, were able to reduce the number of TUNEL-positive apoptotic cells, upregulate the expressions of PCNA and Bcl-2, and downregulate the expression of Bax in retinal tissues of MNU-induced mouse model (Fig. 6C-E). Moreover, both scotopic and photopic ERG analysis demonstrated that the application of Hyp-sEVs^shGAP43^ exhibited limited effects to alleviate MNU-triggered retinal dysfunction (Fig. 6F-J). Moreover, increased number of S-opsin-positive cone photoreceptors in retinal tissues after Hyp-sEVs treatment was reversed by GAP43 knockdown (Fig. 6K). Collectively, these findings provided in vivo evidence that GAP43 was required for Hyp-sEVs-mediated therapeutic effects on MNU-induced retinal photoreceptor injury.

### Hypoxic preconditioning-induced HIF-1α activation inhibited TRIM25-mediated GAP43 ubiquitination and degradation

**Fig. 7:**
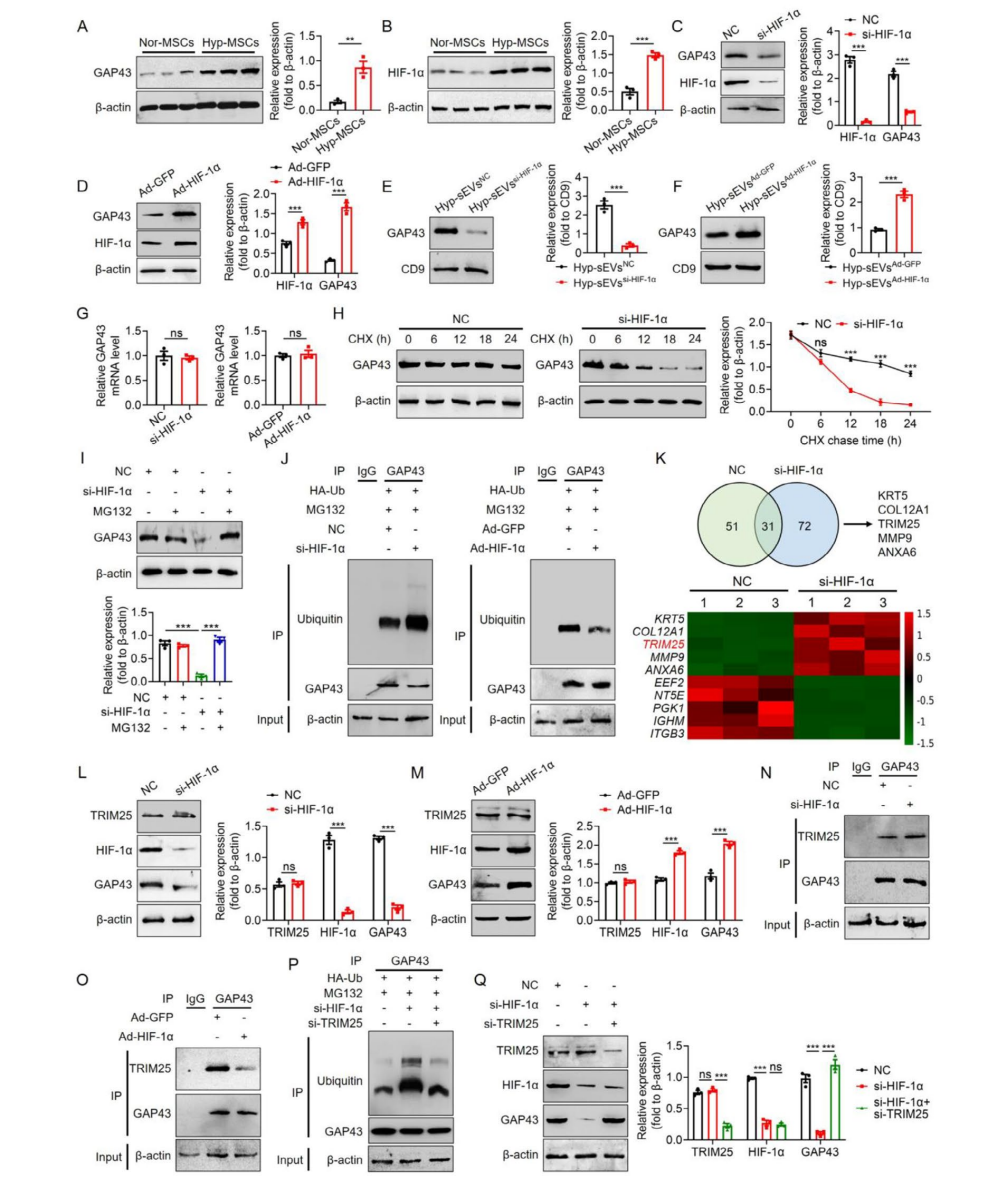
HIF-1α activation prevented TRIM25-mediated GAP43 ubiquitination and degradation in Hyp-MSCs. (**A**, **B**) Western blot for the expressions of GAP43 and HIF-1α in Nor-MSCs and Hyp-MSCs. (**C**, **D**) Western blot for the expressions of HIF-1α and GAP43 in Hyp-MSCs transfected with HIF-1α siRNA and HIF-1α overexpressing plasmid. (**E**, **F**) Western blot for the expression of GAP43 in HIF-1α siRNA-transfected Hyp-sEVs (Hyp-sEVs^si−HIF−1α^) and HIF-1α overexpressing plasmid-transfected Hyp-sEVs (Hyp-sEVs^Ad−HIF−1α^). (**G**) qRT-PCR for the relative GAP43 mRNA level in Hyp-MSCs transfected with HIF-1α siRNA and HIF-1α overexpressing plasmid. (**H**) CHX assay for the half-time of GAP43. (**I**) The effect of MG132 on GAP43 expression. (**J**) Co-IP assay for the ubiquitination level of GAP43. (**K**) LC-MS/MS analysis for the interacting proteins of GAP43. (**L**, **M**) Western blot for the expression of TRIM25 in Hyp-MSCs transfected with HIF-1α siRNA and HIF-1α overexpressing plasmid. (**N**, **O**) Co-IP assay for the binding of GAP43 to TRIM25 in Hyp-MSCs transfected with HIF-1α siRNA and HIF-1α overexpressing plasmid. (**P**) Co-IP assay for the ubiquitination level of GAP43 in Hyp-MSCs transfected with HIF-1α siRNA and TRIM25 siRNA. (**Q**) Western blot for the expression of GAP43 in Hyp-MSCs transfected with HIF-1α siRNA and TRIM25 siRNA. All data are presented as means ± SEM. ns, not significant, **P < 0.01 and ***P < 0.001

Subsequently, we explored the molecular mechanism underlying the enrichment of GAP43 in Hyp-sEVs. Due to the close correlation of the cargo composition in sEVs with donor cells [18], we evaluated GAP43 expression in MSCs under normoxic or hypoxic conditions and verified the increased GAP43 protein level in Hyp-MSCs (Fig. 7A). It is recognized that HIF-1α is an essential factor to regulate cellular processes under hypoxic condition [19]. Compared with Nor-MSCs group, increased expression of HIF-1α in Hyp-MSCs was observed (Fig. 7B). Therefore, we investigated whether HIF-1α is involved in the regulation of GAP43. The results of Western blot showed that HIF-1α siRNA transfection-induced HIF-1α inhibition reduced the protein expression of GAP43 and HIF-1α overexpression resulted in the increased protein level of GAP43 (Fig. 7C, D). Consistently, HIF-1α knockdown and activation in Hyp-MSCs could affect the GAP43 expression in Hyp-sEVs (Fig. 7E, F). However, HIF-1α knockdown and activation in Hyp-MSCs did not markedly alter the GAP43 mRNA level (Fig. 7G), implying that HIF-1α may regulate the stability of GAP43 protein. To substantiate this assumption, we transfected Hyp-MSCs with HIF-1α siRNA and detected the half-life of GAP43 protein after protein synthesis inhibitor cycloheximide (CHX) treatment. Compared with control group, the half-life of GAP43 protein in HIF-1α siRNA transfection group was shorter (Fig. 7H). We then used the proteasome inhibitor MG132 to evaluate the effect of HIF-1α on GAP43 protein degradation. As shown in Fig. 7I, HIF-1α knockdown-induced reduction of GAP43 protein was restored by MG132. The results of Co-IP assay also demonstrated that HIF-1α knockdown enhanced the ubiquitination modification of GAP43, and HIF-1α overexpression reduced the ubiquitinated GAP43 protein level (Fig. 7J). These data suggested that HIF-1α regulated GAP43 protein stability mainly through the ubiquitin-proteasome pathway.

To identify the E3 ligase that mediates GAP43 ubiquitination, we detected the GAP43-binding proteins by Co-IP assay and LC-MS/MS analysis. The results revealed the presence of E3 ubiquitin ligase TRIM25 in GAP43 immunoprecipitation (Fig. 7K). Western blot analysis showed that the changed expression of HIF-1α exerted no significant impact on TRIM25 protein level in Hyp-MSCs (Fig. 7L, M). However, HIF-1α knockdown promoted the binding of GAP43 to TRIM25, and HIF-1α overexpression prevented the interaction between GAP43 and TRIM25 (Fig. 7N, O). To further validate the role of TRIM25 in HIF-1α-mediated regulation of GAP43, we co-transfected Hyp-MSCs with HIF-1α siRNA and TRIM25 siRNA. The results of Co-IP assay and Western blot showed that TRIM25 inhibition reversed the HIF-1α knockdown-induced GAP43 ubiquitination and degradation (Fig. 7P, Q). Collectively, these findings indicated that hypoxic condition-induced HIF-1α activation inhibited TRIM25-mediated GAP43 ubiquitination and degradation in Hyp-MSCs, leading to the production of Hyp-sEVs with high expression of GAP43.

## Discussion

Photoreceptor apoptosis is an important pathological feature of degenerative retinal diseases [20]. Current therapeutic options provide limited benefits. In the present study, we found that the intravitreal injection of Hyp-sEVs effectively inhibited photoreceptor apoptosis and ameliorated retinal degeneration mainly through the delivery of GAP43. Mechanistically, hypoxic stimulation-induced HIF-1α upregulation prevented the TRIM25-mediated ubiquitination modification of GAP43 in Hyp-MSCs, resulting in the enrichment of GAP43 in Hyp-sEVs.

Despite the different and multifactorial etiologies of various retinal disorders, their cause of visual impairment and blindness is photoreceptor loss [21]. In the past decades, MSC-based cell therapy has achieved encouraging progress to alleviate many retinal diseases including photoreceptor deficiency by replacing injured retinal cells [22]. However, the adverse reactions and ocular complications caused by MSC transplantation limit its wide application [23]. Recent studies suggest that MSCs promote tissue regeneration mainly through the paracrine effect [24]. MSC-sEVs exhibit extensive efficient to ameliorate retinal ischemic, inflammation, oxidative damage, and neovascularization [25–28]. Notably, MSC-sEVs treatment represents a promising cell-free strategy to protect retinal photoreceptors from apoptosis, while some weaknesses such as low yield, heterogeneity, and unstable curative effect of MSC-sEVs have not been solved [29, 30]. It is recognized that the therapeutic potential of MSC-sEVs is largely dependent on the status of MSCs [31]. The optimization of culture condition can affect the cellular function of MSCs, thus further enhancing the release and biological activity of MSC-sEVs. Previous evidence has indicated that the oxygen tension of cells in the body is lower than that in the atmosphere [32]. Hypoxic stimulation can upregulate stemness genes in MSC and improve the cellular viability and genetic stability [33]. In this study, we observed that Hyp-MSCs showed elevated proliferation and multidirectional differentiation abilities and released more sEVs compared with Nor-MSCs, which confirms that hypoxic preconditioning enhances MSC activity and increases the yield of MSC-sEVs. Accumulating studies have revealed the enhanced therapeutic effects of Hyp-sEVs in several ocular diseases. For instance, relative to Nor-sEVs, Hyp-sEVs further restore intraocular pressure and retinal shrinkage in a rat model of glaucoma [34]. Hyp-sEVs markedly promote retinal ganglion cell regeneration to alleviate optic nerve compression injury [35]. Here, we found that the localization of Hyp-sEVs was primarily in the ONL after intravitreal injection and for the first time revealed that Hyp-sEVs exerted more effective roles to improve retinal structure and thickness, restore retinal function, and inhibit photoreceptor apoptosis compared with Nor-sEVs, which therefore provides a foundation for developing novel therapy for retinal degeneration.

MSC-sEVs contain complex cargos which contribute to the functional regulation of recipient cells [36]. Shuttle of proteins has been widely proved as the important mechanism underlying MSC-sEVs-mediated protective effects for ocular disorders. Gao et al. report that MSC-sEVs-delivered brain-derived neurotrophic factor (BDNF) accelerates the retrodifferentiation and proliferation of Müller cells by activating Wnt pathway [37]. Moreover, MSC-sEVs attenuate retinal apoptosis and oxidative injury in streptozotocin-induced diabetic rats by transporting neuronal precursor cell-expressed developmentally downregulated 4 (NEDD4) [38]. Recent evidence has also indicated that sEVs-induced protein transfer can regulate retinal tissue homeostasis [39]. Several proteins carried by circulating sEVs are promising candidates to predict the progression of retinal diseases [40]. There is a significant heterogeneity in the ingredients of sEVs from cells cultured in different conditions. In this study, we analyzed the protein composition of Nor-sEVs and Hyp-sEVs by LC-MS/MS, and identified that GAP43 was significantly upregulated in Hyp-sEVs. GAP43 has been previously documented as an intrinsic determinant of axonal growth, regeneration, and plasticity [41]. In retinal system, the activation of GAP43 is closely related to the increased regenerative potential of injured retinal ganglion cells [42]. It is reported that GAP43 accumulates in retinal outer photoreceptor segments of rats kept in the darkness for 10 days [43]. However, the involvement of GAP43 in the restoration of photoreceptor viability is largely unknown. Here, we observed that Hyp-sEVs treatment could reverse the MNU-induced decreased expression of GAP43 in retinal tissues and 661 W cells. Importantly, the knockdown of GAP43 impaired the therapeutic role of Hyp-sEVs to improve retinal degeneration and inhibit photoreceptor apoptosis in vivo and in vitro, thus confirming that GAP43 is a critical mediator of the repairing effects of Hyp-sEVs on retinal injury. The detailed mechanism of GAP43 in regulating photoreceptor function remains to be further explored in future studies.

During hypoxic stimulation, due to the activity inhibition of Prolyl-4-hydroxylase domain (PHD) proteins, HIF-1α is protected against the Von Hippel-Lindau (VHL)-mediated degradation [44]. Accumulating evidence suggests that HIF-1α serves as a master regulator of the response to low oxygen and plays a crucial role in the signal transduction and cellular function alteration. Luo et al. report that hypoxic pretreatment-induced HIF-1α activation improves the survival of MSCs after transplantation for spinal cord injury therapy [45]. The results of Liu et al. show that hypoxic preconditioning enhances the miR-126 level in MSC-sEVs through the upregulation of HIF-1α [46]. However, whether HIF-1α contributes to the enrichment of GAP43 in Hyp-sEVs is still unclear. Herein, our findings revealed that HIF-1α knockdown and overexpression could affect the protein level of GAP43 both in Hyp-MSCs and Hyp-sEVs. CHX and MG132 assays further demonstrated that HIF-1α regulated GAP43 protein stability mainly through the ubiquitin-proteasome manner. Previous studies have shown that fatty acylation and phosphorylation are two major mechanisms for the post-translational modification of GAP43 [47]. Although GAP43 has been also reported as a substrate of the ubiquitin-proteasome system [48], little is known about the E3 ligase to mediate the degradation of GAP43. In this study, we found that TRIM25 was abundant in the GAP43 immunoprecipitation of MSCs transfected with HIF-1α siRNA through mass spectrometry analysis. TRIM25 knockdown reversed the HIF-1α inhibition-induced elevated GAP43 ubiquitination, which suggests that HIF-1α upregulates GAP43 protein expression by inhibiting TRIM25-mediated GAP43 ubiquitination and degradation.

A single injection of sEVs shows effective retinal therapeutic potential in many animal models with retinal injury. For instance, Zhu et al. reveal that a single intravitreal injection of Schwann cell-derived sEVs can mitigate the degeneration of retinal ganglion cells in an optic nerve crush model [49]. In this study, our results also indicated that a single injection of Hyp-sEVs prevented retinal photoreceptor loss in MNU-induced retinal degeneration. However, the retention time of sEVs varies in the different retinal cells [50]. Most fluorescent labeled-sEVs exhibit a significant reduction within two weeks in retinal cells in vivo [51]. Therefore, successive injection may be required to maintain the therapeutic effect of sEVs on chronic retinal injury. Mead et al. report that monthly MSC-sEVs intervention provides retinal neuroprotective effects and ameliorates axon degeneration in a chronic model of ocular hypertension [52]. Xu et al. demonstrate that continuous injection of MSC-sEVs for 4 weeks exerts retinal anti-inflammatory and anti-apoptotic roles in diabetic rats [53]. Recently, the preparation of engineered sEVs is considered a promising approach to enhance their protective value and targeting potential [54]. The status improvement of source cells and direct membrane and content modification of sEVs are two major engineering strategies [55]. The results of Reddy et al. indicate that Bevacizumab-loaded sEVs reduce the frequency of intravitreal injection required for treating diabetic retinopathy [56]. Bao et al. report that degradable poly microcapsules encapsulating MSC-sEVs can increase the retinal residence time of MSC-sEVs for the treatment of retinal ischaemia-reperfusion injury [57]. In this study, we found that hypoxic preconditioning enhanced the effectiveness of MSC-sEVs in photoreceptor protection. Although sEVs-based cell-free strategy has shown therapeutic potential in various retinal diseases, further investigations are still essential to improve the retinal repair efficiency of sEVs and reduce their injection frequency.

## Conclusions

In summary, we demonstrate that Hyp-sEVs exhibit enhanced therapeutic potential to ameliorate retinal structural and functional degeneration and inhibit photoreceptor apoptosis by delivering enriched GAP43 compared with Nor-sEVs. Hypoxic preconditioning-induced HIF-1α activation prevents TRIM25-mediated GAP43 ubiquitination and degradation, resulting in the upregulation of GAP43 in Hyp-sEVs. Therefore, our findings provide new insights into the mechanism underlying the retinal protective effects of Hyp-sEVs and highlight an optimized cell-free strategy for retinal degeneration therapy.

### Electronic supplementary material

Below is the link to the electronic supplementary material.

Supplementary Material 1

## Data Availability

All data in this study are available from the corresponding author upon reasonable request.

## Abbreviations

sEVs: Small extracellular vesicles
MSC: Mesenchymal stem/stromal cell
MSC-sEVs: MSC-derived sEVs
Hyp-sEVs: Hypoxic preconditioned MSC-sEVs
MNU: N-methyl-N-nitrosourea
Nor-sEVs: Normoxic conditioned MSC-sEVs
GAP43: Growth-associated protein 43
HIF-1α: Hypoxia-inducible factor-1α
TRIM25: Tripartite motif-containing protein 25
PBS: Phosphate buffer solution
Nor-MSCs: Normoxic conditioned MSCs
Hyp-MSCs: Hypoxic preconditioned MSCs
FBS: Fetal bovine serum
TEM: Transmission electron microscopy
NTA: NanoSight tracking analysis
TSG101: Tumor susceptibility gene 101
ERG: Electroretinogram
H&E: Hematoxylin and eosin
BSA: Bovine serum albumin
TUNEL: Terminal deoxynucleotidyl transferase dUTP nick end labelling
LC-MS/MS: Liquid chromatography-tandem mass spectrometry
shRNA: Short hairpin RNA
PCNA: Proliferating cell nuclear antigen
Co-IP: Co-immunoprecipitation
ONL: Outer nuclear layer
Hyp-MSCs^shGAP43^: GAP43 shRNA-transfected Hyp-MSCs
Hyp-sEVs^shGAP43^: Hyp-MSCs^shGAP43^-derived sEVs
CHX: Cycloheximide
BDNF: Brain-derived neurotrophic factor
NEDD4: Neuronal precursor cell-expressed developmentally downregulated 4
PHD: Prolyl-4-hydroxylase domain
VHL: Von Hippel-Lindau

## References

[1] Wang Y, Punzo C, Ash JD, Lobanova ES (2022). Tsc2 knockout counteracts ubiquitin-proteasome system insufficiency and delays photoreceptor loss in retinitis pigmentosa. Proc Natl Acad Sci U S A.

[2] Osada H, Toda E, Homma K, Guzman NA, Nagai N, Ogawa M, Negishi K, Arita M, Tsubota K, Ozawa Y (2021). ADIPOR1 deficiency-induced suppression of retinal ELOVL2 and docosahexaenoic acid levels during photoreceptor degeneration and visual loss. Cell Death Dis.

[3] Charish J, Shabanzadeh AP, Chen D, Mehlen P, Sethuramanujam S, Harada H, Bonilha VL, Awatramani G, Bremner R, Monnier PP (2020). Neogenin neutralization prevents photoreceptor loss in inherited retinal degeneration. J Clin Invest.

[4] Botto C, Rucli M, Tekinsoy MD, Pulman J, Sahel JA, Dalkara D (2022). Early and late stage gene therapy interventions for inherited retinal degenerations. Prog Retin Eye Res.

[5] Mahato B, Kaya KD, Fan Y, Sumien N, Shetty RA, Zhang W, Davis D, Mock T, Batabyal S, Ni A, Mohanty S, Han Z, Farjo R, Forster MJ, Swaroop A, Chavala SH (2020). Pharmacologic fibroblast reprogramming into photoreceptors restores vision. Nature.

[6] van Niel G, Carter DRF, Clayton A, Lambert DW, Raposo G, Vader P (2022). Challenges and directions in studying cell-cell communication by extracellular vesicles. Nat Rev Mol Cell Biol.

[7] Jia Y, Yu L, Ma T, Xu W, Qian H, Sun Y, Shi H (2022). Small extracellular vesicles isolation and separation: current techniques, pending questions and clinical applications. Theranostics.

[8] Sanz-Ros J, Romero-García N, Mas-Bargues C, Monleón D, Gordevicius J, Brooke RT, Dromant M, Díaz A, Derevyanko A, Guío-Carrión A, Román-Domínguez A, Inglés M, Blasco MA, Horvath S, Viña J, Borrás C (2022). Small extracellular vesicles from young adipose-derived stem cells prevent frailty, improve health span, and decrease epigenetic age in old mice. Sci Adv.

[9] Tang Y, Kang Y, Zhang X, Cheng C (2023). Mesenchymal stem cell exosomes as nanotherapeutics for dry age-related macular degeneration. J Control Release.

[10] Deng CL, Hu CB, Ling ST, Zhao N, Bao LH, Zhou F, Xiong YC, Chen T, Sui BD, Yu XR, Hu CH (2021). Photoreceptor protection by mesenchymal stem cell transplantation identifies exosomal MiR-21 as a therapeutic for retinal degeneration. Cell Death Differ.

[11] Zhang X, Zhang H, Gu J, Zhang J, Shi H, Qian H, Wang D, Xu W, Pan J, Santos HA (2021). Engineered extracellular vesicles for cancer therapy. Adv Mater.

[12] Yang Y, Lee EH, Yang Z (2022). Hypoxia-conditioned mesenchymal stem cells in tissue regeneration application. Tissue Eng Part B Rev.

[13] Li Q, Xu Y, Lv K, Wang Y, Zhong Z, Xiao C, Zhu K, Ni C, Wang K, Kong M, Li X, Fan Y, Zhang F, Chen Q, Li Y, Li Q, Liu C, Zhu J, Zhong S, Wang J, Chen Y, Zhao J, Zhu D, Wu R, Chen J, Zhu W, Yu H, Ardehali R, Zhang JJ, Wang J, Hu X (2021). Small extracellular vesicles containing mir-486-5p promote angiogenesis after Myocardial Infarction in mice and nonhuman primates. Sci Transl Med.

[14] Hu N, Cai Z, Jiang X, Wang C, Tang T, Xu T, Chen H, Li X, Du X, Cui W (2023). Hypoxia-pretreated ADSC-derived exosome-embedded hydrogels promote angiogenesis and accelerate diabetic wound healing. Acta Biomater.

[15] Liu W, Rong Y, Wang J, Zhou Z, Ge X, Ji C, Jiang D, Gong F, Li L, Chen J, Zhao S, Kong F, Gu C, Fan J, Cai W (2020). Exosome-shuttled miR-216a-5p from hypoxic preconditioned mesenchymal stem cells repair traumatic spinal cord injury by shifting microglial M1/M2 polarization. J Neuroinflammation.

[16] Qiao C, Xu W, Zhu W, Hu J, Qian H, Yin Q, Jiang R, Yan Y, Mao F, Yang H, Wang X, Chen Y (2008). Human mesenchymal stem cells isolated from the umbilical cord. Cell Biol Int.

[17] Tan E, Ding XQ, Saadi A, Agarwal N, Naash MI, Al-Ubaidi MR (2004). Expression of cone-photoreceptor-specific antigens in a cell line derived from retinal tumors in transgenic mice. Invest Ophthalmol Vis Sci.

[18] Gupta D, Zickler AM, El Andaloussi S (2021). Dosing extracellular vesicles. Adv Drug Deliv Rev.

[19] Gunton JE (2020). Hypoxia-inducible factors and Diabetes. J Clin Invest.

[20] Power M, Das S, Schütze K, Marigo V, Ekström P, Paquet-Durand F (2020). Cellular mechanisms of hereditary photoreceptor degeneration - focus on cGMP. Prog Retin Eye Res.

[21] Lee JY, Care RA, Della Santina L, Dunn FA (2021). Impact of photoreceptor loss on retinal circuitry. Annu Rev Vis Sci.

[22] Sharma A, Jaganathan BG (2021). Stem cell therapy for retinal degeneration: the evidence to date. Biologics.

[23] Gagliardi G, Ben M, Barek K, Goureau O (2019). Photoreceptor cell replacement in macular degeneration and retinitis pigmentosa: a pluripotent stem cell-based approach. Prog Retin Eye Res.

[24] Weng Z, Zhang B, Wu C, Yu F, Han B, Li B, Li L (2021). Therapeutic roles of mesenchymal stem cell-derived extracellular vesicles in cancer. J Hematol Oncol.

[25] Mathew B, Acha LG, Torres LA, Huang CC, Liu A, Kalinin S, Leung K, Dai Y, Feinstein DL, Ravindran S, Roth S (2023). MicroRNA-based engineering of mesenchymal stem cell extracellular vesicles for treatment of retinal ischemic disorders: engineered extracellular vesiclesand retinal ischemia. Acta Biomater.

[26] Zhang J, Li P, Zhao G, He S, Xu D, Jiang W, Peng Q, Li Z, Xie Z, Zhang H, Xu Y, Qi L (2022). Mesenchymal stem cell-derived extracellular vesicles protect retina in a mouse model of retinitis pigmentosa by anti-inflammation through miR-146a-Nr4a3 axis. Stem Cell Res Ther.

[27] Ebrahim N, El-Halim HEA, Helal OK, El-Azab NE, Badr OAM, Hassouna A, Saihati HAA, Aborayah NH, Emam HT, El-Wakeel HS, Aljasir M, El-Sherbiny M, Sarg NAS, Shaker GA, Mostafa O, Sabry D, Fouly MAK, Forsyth NR, Elsherbiny NM, Salim RF (2022). Effect of bone marrow mesenchymal stem cells-derived exosomes on diabetes-induced retinal injury: implication of Wnt/ b-catenin signaling pathway. Biomed Pharmacother.

[28] Gu C, Zhang H, Gao Y (2021). Adipose mesenchymal stem cells-secreted extracellular vesicles containing microRNA-192 delays diabetic retinopathy by targeting ITGA1. J Cell Physiol.

[29] Iavorovschi AM, Wang A (2020). Engineering mesenchymal stromal/stem cell-derived extracellular vesicles with improved targeting and therapeutic efficiency for the treatment of central nervous system disorders. Neural Regen Res.

[30] Choi SW, Seo S, Hong HK, Yoon SJ, Kim M, Moon S, Lee JY, Lim J, Lee JB, Woo SJ. Therapeutic extracellular vesicles from tonsil-derived mesenchymal stem cells for the treatment of retinal degenerative Disease. Tissue Eng Regen Med. 2023. 10.1007/s13770-023-00555-8 PMC10519919 37440108

[31] Psaraki A, Ntari L, Karakostas C, Korrou-Karava D, Roubelakis MG (2022). Extracellular vesicles derived from mesenchymal stem/stromal cells: the regenerative impact in Liver Diseases. Hepatology.

[32] Chen S, Sun F, Qian H, Xu W, Jiang J (2022). Preconditioning and engineering strategies for improving the efficacy of mesenchymal stem cell-derived exosomes in cell-free therapy. Stem Cells Int.

[33] Tian Y, Fang J, Zeng F, Chen Y, Pei Y, Gu F, Ding C, Niu G, Gu B (2022). The role of hypoxic mesenchymal stem cells in Tumor immunity. Int Immunopharmacol.

[34] Seong HR, Noh CH, Park S, Cho S, Hong SJ, Lee AY, Geum D, Hong SC, Park D, Kim TM, Choi EK, Kim YB (2023). Intraocular pressure-lowering and retina-protective effects of exosome-rich conditioned media from human amniotic membrane stem cells in a rat model of glaucoma. Int J Mol Sci.

[35] Park M, Shin HA, Duong VA, Lee H, Lew H (2022). The role of extracellular vesicles in optic nerve injury: neuroprotection and mitochondrial homeostasis. Cells.

[36] Boulestreau J, Maumus M, Jorgensen C, Noël D (2021). Extracellular vesicles from mesenchymal stromal cells: therapeutic perspectives for targeting senescence in osteoarthritis. Adv Drug Deliv Rev.

[37] Gao Y, Li H, Qin C, Yang B, Ke Y (2022). Embryonic stem cells-derived exosomes enhance retrodifferentiation of retinal Müller cells by delivering BDNF protein to activate wnt pathway. Immunobiology.

[38] Sun F, Sun Y, Zhu J, Wang X, Ji C, Zhang J, Chen S, Yu Y, Xu W, Qian H (2022). Mesenchymal stem cells-derived small extracellular vesicles alleviate diabetic retinopathy by delivering NEDD4. Stem Cell Res Ther.

[39] Liu J, Jiang F, Jiang Y, Wang Y, Li Z, Shi X, Zhu Y, Wang H, Zhang Z (2020). Roles of exosomes in ocular Diseases. Int J Nanomedicine.

[40] You JX, Qi SN, Fu JL, Wang CG, Su GF (2021). Circulating exosomes in ophthalmic Disease: novel carriers of biological information circulating exosomes in ophthalmic Disease. Eur Rev Med Pharmacol Sci.

[41] Watson DC, Bayik D, Storevik S, Moreino SS, Sprowls SA, Han J, Augustsson MT, Lauko A, Sravya P, Røsland GV, Troike K, Tronstad KJ, Wang S, Sarnow K, Kay K, Lunavat TR, Silver DJ, Dayal S, Joseph JV, Mulkearns-Hubert E, Ystaas LAR, Deshpande G, Guyon J, Zhou Y, Magaut CR, Seder J, Neises L, Williford SE, Meiser J, Scott AJ, Sajjakulnukit P, Mears JA, Bjerkvig R, Chakraborty A, Daubon T, Cheng F, Lyssiotis CA, Wahl DR, Hjelmeland AB, Hossain JA, Miletic H, Lathia JD (2023). GAP43-dependent mitochondria transfer from astrocytes enhances glioblastoma tumorigenicity. Nat Cancer.

[42] Fung JCL, Cho EYP (2020). Methylene blue promotes survival and GAP-43 expression of retinal ganglion cells after optic nerve transection. Life Sci.

[43] López-Costa JJ, Goldstein J, Mangeaud M, Saavedra JP (2001). Expression of GAP-43 in the retina of rats following protracted illumination. Brain Res.

[44] Cowman SJ, Koh MY (2022). Revisiting the HIF switch in the Tumor and its immune microenvironment. Trends Cancer.

[45] Luo Z, Wu F, Xue E, Huang L, Yan P, Pan X, Zhou Y (2019). Hypoxia preconditioning promotes bone marrow mesenchymal stem cells survival by inducing HIF-1α in injured neuronal cells derived exosomes culture system. Cell Death Dis.

[46] Liu W, Li L, Rong Y, Qian D, Chen J, Zhou Z, Luo Y, Jiang D, Cheng L, Zhao S, Kong F, Wang J, Zhou Z, Xu T, Gong F, Huang Y, Gu C, Zhao X, Bai J, Wang F, Zhao W, Zhang L, Li X, Yin G, Fan J, Cai W (2020). Hypoxic mesenchymal stem cell-derived exosomes promote bone fracture healing by the transfer of miR-126. Acta Biomater.

[47] Chung D, Shum A, Caraveo G (2020). GAP-43 and BASP1 in axon regeneration: implications for the treatment of neurodegenerative Diseases. Front Cell Dev Biol.

[48] De Moliner KL, Wolfson ML, Perrone Bizzozero N, Adamo AM (2005). Growth-associated protein-43 is degraded via the ubiquitin-proteasome system. J Neurosci Res.

[49] Zhu S, Chen L, Wang M, Zhang J, Chen G, Yao Y, Song S, Li T, Xu S, Yu Z, Shen B, Xu D, Chi ZL, Wu W (2023). Schwann cell-derived extracellular vesicles as a potential therapy for retinal ganglion cell degeneration. J Control Release.

[50] Mathew B, Ravindran S, Liu X, Torres L, Chennakesavalu M, Huang CC, Feng L, Zelka R, Lopez J, Sharma M, Roth S (2019). Mesenchymal stem cell-derived extracellular vesicles and retinal ischemia-reperfusion. Biomaterials.

[51] Mathew B, Torres LA, Gamboa Acha L, Tran S, Liu A, Patel R, Chennakesavalu M, Aneesh A, Huang CC, Feinstein DL, Mehraeen S, Ravindran S, Roth S (2021). Uptake and distribution of administered bone marrow mesenchymal stem cell extracellular vesicles in retina. Cells.

[52] Mead B, Ahmed Z, Tomarev S (2018). Mesenchymal stem cell-derived small extracellular vesicles promote neuroprotection in a genetic DBA/2J mouse model of glaucoma. Invest Ophthalmol Vis Sci.

[53] Xu Z, Tian N, Li S, Li K, Guo H, Zhang H, Jin H, An M, Yu X (2021). Extracellular vesicles secreted from mesenchymal stem cells exert anti-apoptotic and anti-inflammatory effects via transmitting microRNA-18b in rats with diabetic retinopathy. Int Immunopharmacol.

[54] Richter M, Vader P, Fuhrmann G (2021). Approaches to surface engineering of extracellular vesicles. Adv Drug Deliv Rev.

[55] Pan Z, Sun W, Chen Y, Tang H, Lin W, Chen J, Chen C (2022). Extracellular vesicles in tissue engineering: biology and engineered strategy. Adv Healthc Mater.

[56] Reddy SK, Ballal AR, Shailaja S, Seetharam RN, Raghu CH, Sankhe R, Pai K, Tender T, Mathew M, Aroor A, Shetty AK, Adiga S, Devi V, Muttigi MS, Upadhya D (2023). Small extracellular vesicle-loaded bevacizumab reduces the frequency of intravitreal injection required for diabetic retinopathy. Theranostics.

[57] Bao H, Tian Y, Wang H, Ye T, Wang S, Zhao J, Qiu Y, Li J, Pan C, Ma G, Wei W, Tao Y. Exosome-loaded degradable polymeric microcapsules for the treatment of vitreoretinal Diseases. Nat Biomed Eng. 2023. 10.1038/s41551-023-01112-3 37872369

