# Supplementary material for: Hypoxic preconditioned MSCs-derived small extracellular vesicles for photoreceptor protection in retinal degeneration
Source: J Nanobiotechnology. 2023 Nov 25;21:449. doi: 10.1186/s12951-023-02225-2 (PMC10675959; doi:10.1186/s12951-023-02225-2)
Supplement: Supplementary file 1 — Supplementary Material 1 [file 12951_2023_2225_MOESM1_ESM.docx]

**Supplementary material for**

**Hypoxic preconditioned MSCs-derived small extracellular vesicles for** **photoreceptor protection in retinal degeneration**

Yuntong Sun^1†^, Yuntao Sun^2†^, Shenyuan Chen^2†^, Yifan Yu^2^, Yongjun Ma^1^* and Fengtian Sun^1^*

^1^Department of Clinical Laboratory, Affiliated Jinhua Hospital, Zhejiang University School of Medicine, Jinhua 321000, Zhejiang, China

^2^Jiangsu Province Key Laboratory of Medical Science and Laboratory Medicine, Department of Laboratory Medicine, School of Medicine, Jiangsu University, Zhenjiang 212013, Jiangsu, China

^†^Yuntong Sun, Yuntao Sun and Shenyuan Chen contributed equally to this work.

*Correspondence: Fengtian Sun (jsdxsft@163.com); Yongjun Ma (jhmyj71@sina.cn).


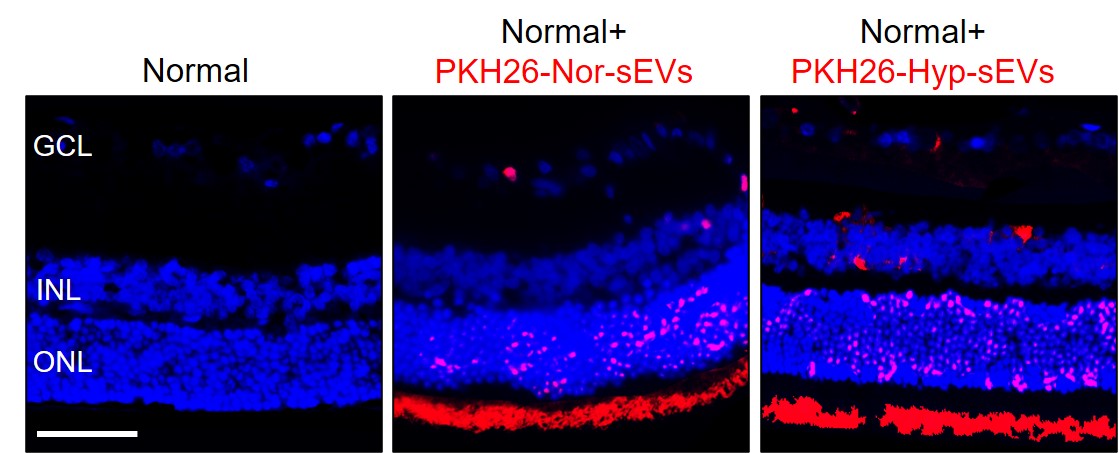


**Fig. S1.** Tracing of PKH26-labeled sEVs in retina tissues of normal mice after intravitreal injection for 24 h. Scale bars, 50 μm. GCL, ganglion cell layer; INL, inner nuclear layer; ONL, outer nuclear layer.
